# Aortopulmonary collaterals in single ventricle: incidence, associated factors and clinical significance

**DOI:** 10.1093/icvts/ivac190

**Published:** 2022-07-25

**Authors:** Melvin Schmiel, Takashi Kido, Stanimir Georgiev, Melchior Burri, Paul Philipp Heinisch, Janez Vodiskar, Martina Strbad, Peter Ewert, Alfred Hager, Jürgen Hörer, Masamichi Ono

**Affiliations:** Department of Congenital and Pediatric Heart Surgery, German Heart Center Munich, Technische Universität München, Munich, Germany; Division of Congenital and Pediatric Heart Surgery, University Hospital of Munich, Ludwig-Maximilians-Universität, Munich, Germany; Department of Congenital and Pediatric Heart Surgery, German Heart Center Munich, Technische Universität München, Munich, Germany; Division of Congenital and Pediatric Heart Surgery, University Hospital of Munich, Ludwig-Maximilians-Universität, Munich, Germany; Department of Pediatric Cardiology and Congenital Heart Disease, German Heart Center Munich, Technical University of Munich, Munich, Germany; Department of Cardiovascular Surgery, German Heart Center Munich, Technische Universität München, Munich, Germany; Department of Congenital and Pediatric Heart Surgery, German Heart Center Munich, Technische Universität München, Munich, Germany; Division of Congenital and Pediatric Heart Surgery, University Hospital of Munich, Ludwig-Maximilians-Universität, Munich, Germany; Department of Congenital and Pediatric Heart Surgery, German Heart Center Munich, Technische Universität München, Munich, Germany; Division of Congenital and Pediatric Heart Surgery, University Hospital of Munich, Ludwig-Maximilians-Universität, Munich, Germany; Department of Congenital and Pediatric Heart Surgery, German Heart Center Munich, Technische Universität München, Munich, Germany; Division of Congenital and Pediatric Heart Surgery, University Hospital of Munich, Ludwig-Maximilians-Universität, Munich, Germany; Department of Pediatric Cardiology and Congenital Heart Disease, German Heart Center Munich, Technical University of Munich, Munich, Germany; Department of Pediatric Cardiology and Congenital Heart Disease, German Heart Center Munich, Technical University of Munich, Munich, Germany; Department of Congenital and Pediatric Heart Surgery, German Heart Center Munich, Technische Universität München, Munich, Germany; Division of Congenital and Pediatric Heart Surgery, University Hospital of Munich, Ludwig-Maximilians-Universität, Munich, Germany; Department of Congenital and Pediatric Heart Surgery, German Heart Center Munich, Technische Universität München, Munich, Germany; Division of Congenital and Pediatric Heart Surgery, University Hospital of Munich, Ludwig-Maximilians-Universität, Munich, Germany

**Keywords:** Aortopulmonary collateral arteries, Bidirectional cavopulmonary shunt, Total cavopulmonary connection, Hypoplastic left heart syndrome, Norwood procedure

## Abstract

**OBJECTIVES:**

Clinical significance of aortopulmonary collaterals (APCs) in patients with univentricular heart remains controversial. This study aimed to evaluate the incidence and associated factors for APCs and their influence during staged palliation.

**METHODS:**

In total, 430 patients who underwent staged palliation by bidirectional Glenn shunt and total cavopulmonary connection between 2003 and 2019 were examined. APCs were determined by angiogram. Incidence and interventions for APCs were analysed.

**RESULTS:**

The most frequent diagnosis was hypoplastic left heart syndrome in 146 (34%) patients. The median age at Glenn and Fontan was 4.9 months and 2.1 years, respectively. APCs were observed in 54 (13%) patients at Glenn and in 179 (42%) at Fontan. Closure of APCs was performed before Glenn in 12 (3%) patients, at Glenn in 13 (3%), after Glenn in 8 (2%), before Fontan in 44 (10%), at Fontan in 26 (6%) and after Fontan in 52 (12%). Hypoplastic left heart syndrome (*P* < 0.01) was highly associated with the development of APCs before Glenn. Lower Nakata-Index and younger age at Glenn shunt were associated with the development of APCs at Fontan procedure. The presence of APCs or intervention for APCs before total cavopulmonary connection did not influence intensive care unit stay or mortality after total cavopulmonary connection.

**CONCLUSIONS:**

APCs were most frequently observed before Fontan procedure. Hypoplastic left heart syndrome was highly associated with the development of APCs before Glenn shunt. Lower Nakata-Index and younger age at Glenn shunt were associated with APCs before Fontan procedure.

**Figure:**
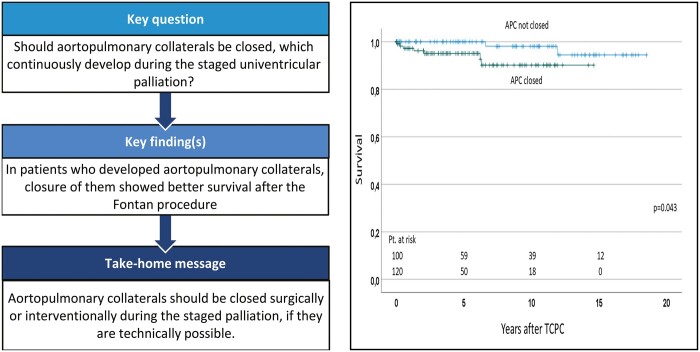

## INTRODUCTION

Aortopulmonary collaterals (APCs) represent an unpredictable source of pulmonary blood flow (PBF) in patients with univentricular heart and are observed in each stage of surgical reconstruction towards Fontan circulation, and also after the Fontan completion [1, 2]. Theoretically, APCs result in volume loading of the systemic ventricle, while simultaneously increasing the PBF and pulmonary artery pressure. Before TCPC, they might enhance arterial oxygen saturation to mitigate the cyanosis of the patients. Early studies before 2001 demonstrated controversial results about the influence of APCs for clinical significance [3–7]. Recent studies using cardiac magnetic resonance (CMR) imaging techniques have enabled researchers to quantify the amount of APC flow [8–14]. Results demonstrated that APC flow was highest in pre-Fontan stage and decreased after the Fontan completion [9, 10]. Studies with CMR have also postulated the evidence that the amount of APC flow correlated with adverse outcomes such as prolonged pleural effusions and longer hospital stay after the Fontan procedure [11–14]. As for therapeutic aspects, many centres aggressively abolish or decrease the APC flow using embolization techniques, reporting therapeutic effects of coil embolization of APCs [15–17]. However, the clinical significance of coil embolization on late outcome is not fully known, and factors for the development of APCs have not been entirely clarified [1, 8, 10]. Furthermore, the impact of APCs on the development of pulmonary arteries and pulmonary artery pressure has been scarcely investigated.

In the present study, we review our large cohort of clinical experiences of patients with univentricular heart who accomplished staged surgical reconstruction by means of bidirectional cavopulmonary shunt (BCPS) and total cavopulmonary connection (TCPC). We evaluated the incidence of APCs and catheter embolization in each stage of surgical palliation. We also aimed to identify factors associated with the development of APCs and to determine the significance of APCs for postoperative outcome after TCPC.

## MATERIALS AND METHODS

### Ethical statement

The Institutional Review Board of the Technical University of Munich approved the study (approved number of 305/20 S-KH on 2 June 2020). Because of the retrospective and observational nature of the study, the need for individual patient consent was waived.

### Patients

**Figure 1:**
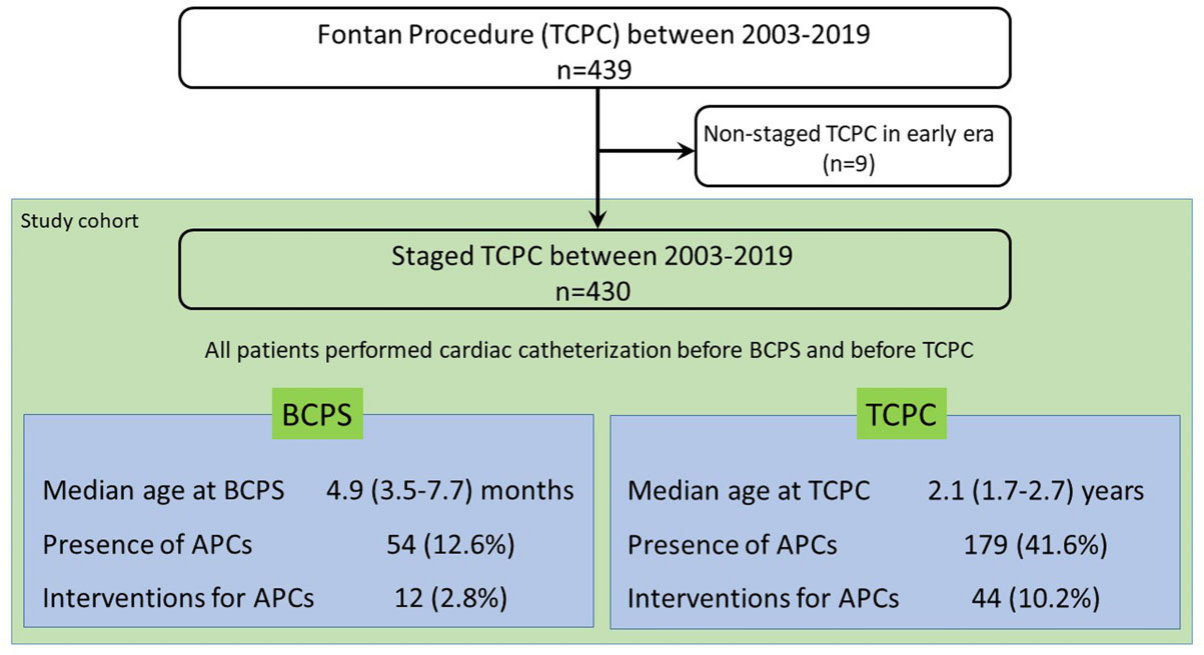
Flow diagram showing patient enrolment and profile. Age at BCPS and TCPC was shown with Median and interquartile ranges. BCPS: bidirectional cavopulmonary shunt; TCPC: total cavopulmonary connection.

We reviewed the medical records of 439 patients with univentricular heart who underwent TCPC at the German Heart Center Munich between 2003 and 2019, and 9 patients who underwent non-staged TCPC (patients who had no previous BCPS) were excluded from this study. Therefore, a total of 430 patients who underwent TCPC as a staged procedure were included in this study (Fig. 1). Patient diagnosis of functional univentricular heart was classified according to the modified Congenital Heart Surgery Nomenclature and Database Project classification [18].

### Surgical strategy and operative techniques

BCPS was performed using standard cardiopulmonary bypass, and antegrade PBF was eliminated [19, 20]. When there was pulmonary stenosis, it was augmented using pericardial patch, and Glenn anastomosis was performed on the augmented patch, or between the native pulmonary artery and the patch. Fontan completion by means of extracardiac TCPC was performed using a Gore-Tex tube graft. Fenestration was created in only a few patients with high risk [21, 22]. Surgical closure of APCs at the time of operation included right internal mammary artery (RIMA) and/or left internal mammary artery (LIMA) closure. After the median sternotomy, RIMA and/or LIMA was prepared, closed and divided using clips at the most proximal and most distal site.

### Angiographic detection of aortopulmonary collaterals and haemodynamic evaluation

The presence of APCs was determined by the finding of angiogram [1, 3–5]. Pulmonary angiograms were reviewed for areas lacking antegrade perfusion (‘washout’) from all patients. Then, selective angiography of the brachiocephalic, subclavian and internal thoracic arteries was performed. Aortography was also performed. We defined APCs as one arising from the arterial circulation if they had a discretely identifiable angiographic origin, supplied the pulmonary parenchyma and opacified the pulmonary arteries or veins, or both. Interventional coil embolization of APCs was performed when they were technically and clinically feasible. The diameter of each identifiable collateral vessel was estimated by comparison of the vessel diameter to the known diameter of catheters presented in the angiogram. Angiograms in which collateral vessels were visualized but which were ambiguous with respect to their origin or distribution of blood flow were not included in the analysis. Nakata-Index was calculated using pulmonary angiograms according to the report from Nakata *et al.* [23]. Measurement of the degree of atrioventricular valve regurgitation and systemic ventricular function by echocardiogram was reported in our previous study [24].

### Follow-up data

The patients obtained outpatient follow-up with paediatric cardiologists, and follow-up times were defined per patient as the time from the TCPC to the time of the last visit. For patients who died, censoring occurred at the time of death.

### Statistical analysis

Categorical variables are presented as absolute numbers and percentages. Continuous variables are expressed as means (standard deviations) or medians with interquartile ranges, if appropriate. An independent Student’s *t*-test was used to compare normally distributed variables. The Mann–Whitney *U*-test was used for variables that were not normally distributed. Factors associated with the development of APCs before BCPS and before TCPC were identified using a logistic regression analysis. The presence of APCs was defined as dependent variable. Factors potentially associated with the development of APCs were tested as a covariant. Variables analysed in this study are shown in Supplementary Material, Table S1. Those variables with a *P*-value <0.1 in univariable analysis were considered for entry into the multivariable models. *P*-values <0.05 were considered significant. Survival after the TCPC was calculated using the Kaplan–Meier method and comparison with and without APCs was performed using log-rank test. Analysis for mortality following TCPC was performed using Cox regression model. Data analysis and graphing were performed with the Statistical Package for the Social Sciences (SPSS) version 25.0 for Windows (IBM, Ehningen, Germany).

## RESULTS

Baseline characteristics of the study cohort are displayed in Table 1. In brief, the most frequent diagnosis was hypoplastic left heart syndrome (HLHS, *n* = 143, 33%). Dominant right ventricle was observed in 256 (60%) patients. The median age at BCPS and TCPC was 4.9 (interquartile ranges 3.5–7.7) months and 2.1 (1.7–2.7) years, respectively.

**Table 1:** Baseline characteristics of patients

Variables		*N* (%) or median (IQR)
Number of patients		430
Male sex		267 (62.1)
Primary diagnosis		
	Hypoplastic left heart syndrome (HLHS)	146 (34.0)
	Univentricular heart (UVH)	92 (21.4)
	Tricuspid atresia (TA)	64 (14.9)
	Double inlet left ventricle (DILV)	44 (10.2)
	Unbalanced atrioventricular septal defect (UAVSD)	16 (3.7)
	Pulmonary atresia and intact ventricular septum (PAIVS)	24 (5.6)
	Congenitally corrected transposition of the great arteries (ccTGA)	20 (4,7)
	Other variants	24 (5.6)
Heterotaxy syndrome		33 (7.7)
Dextrocardia		36 (8.4)
Dominant right ventricle		256 (59.5)
Associated cardiac anomaly		
	Transposition of the great arteries (TGA)	120 (27.9)
	Double outlet right ventricle (DORV)	52 (12.1)
	Coarctation of the aorta (CoA)	57 (13.3)
	Anomalous pulmonary venous drainage	31 (7.2)
	Anomalous systemic venous drainage	45 (10.5)
	Common atrioventricular valve (CAVV)	44 (10.2)
Stage I procedure		
	Norwood procedure	218 (50.7)
	Sano shunt	93 (21.6)
	Systemic-to-pulmonary shunt (SPS)	127 (29.6)
	Pulmonary artery banding (PAB)	43 (10.0)

IQR: interquartile ranges.

### Incidence and origin of aortopulmonary collaterals

The incidence of APCs at each stage of surgical reconstruction is shown in Table 2. Totally, APCs were observed in 220 patients (52%). APCs were found in 54 patients (13%) before BCPS, in 179 (42%) before TCPC, respectively. The origin of APCs in each stage is shown in Table 2 and Supplementary Material, Fig. S1. Before BCPS, the RIMA (*n* = 49, 91%) was the most frequent origin of APCs, followed by LIMA (*n* = 22, 41%), descending aorta (*n* = 7, 13%), right subclavian artery (*n* = 5, 9%) and left subclavian artery (*n* = 1, 2%). The diameter of APCs from each origin, including RIMA, LIMA, right subclavian artery, left subclavian artery and descending aorta, is shown in Supplementary Material, Fig. S2. Pulmonary arterio-venous malformations (PAVMs) were observed before TCPC in seven patients. The details of these patients were demonstrated in Supplementary Material, Table S2.

**Table 2:** Incidence and origin of APCs

Variables	*N* (%)
Number of patients	430
Total	220 (51.2)
Pre-BCPS	54 (12.6)
Laterality	
Right	54 (100.0)
Left	37 (68.5)
Origin	
RIMA	49 (90.7)
LIMA	22 (40.7)
RSCA	5 (9.3)
LSCA	1 (1.9)
Descending aorta	7 (13.0)
Pre-TCPC	179 (41.6)
Laterality	
Right	165 (92.2)
Left	123 (68.7)
Origin	
RIMA	159 (88.8)
LIMA	133 (74.3)
RSCA	43 (24.0)
LSCA	20 (11.2)
Descending aorta	15 (8.4)
After TCPC	70 (16.3)
Laterality	
Right	65 (92.9)
Left	53 (75.7)
Origin	
RIMA	37 (52.9)
LIMA	31 (44.3)
RSCA	21 (30.0)
LSCA	17 (24.6)
Descending aorta	6 (8.6)

APCs: aortopulmonary collaterals; BCPS: bidirectional cavopulmonary shunt; LIMA: left internal mammary artery; LSCA: left subclavian artery; RIMA: right internal mammary artery; RSCA: right subclavian artery; TCPC, total cavopulmonary connection.

### Interventions

**Figure 2:**
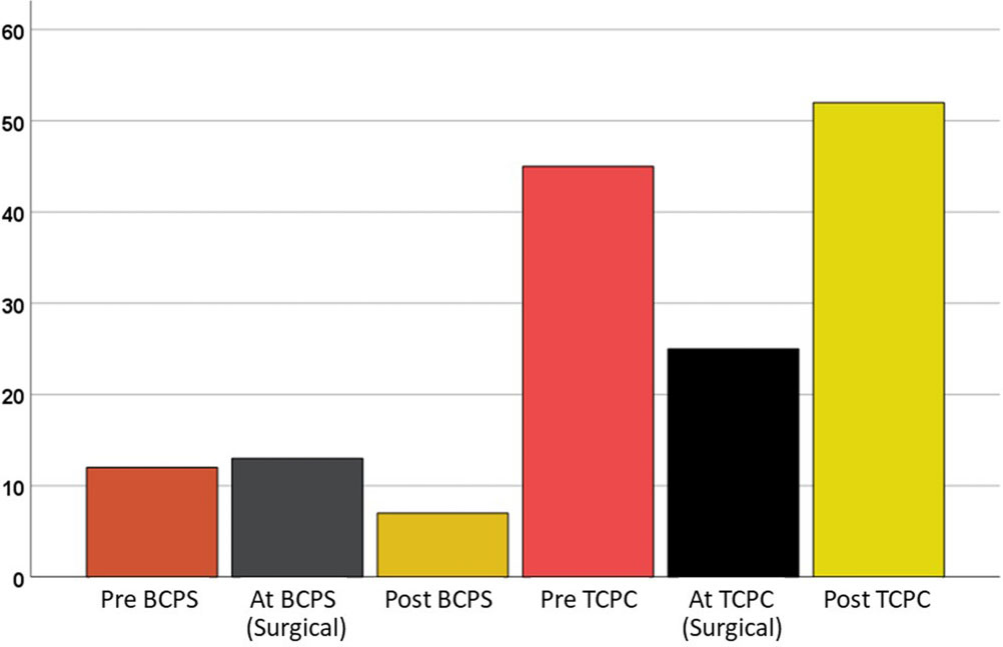
Survival after TCPC comparing the patients who closed APCs and those who did not. APCs: aortopulmonary collaterals; TCPC: total cavopulmonary connection.

Totally, interventional closure of APCs was performed in 120 (28%) patients (Table 3). As for the timing of APCs closure, it was performed before BCPS in 12 (3%) patients, after BCPS in 8 (2%) patients, before TCPC in 44 (10%) patients, and after TCPC in 52 (12%) patients. Surgical closure of APCs was performed in 13 (3%) patients at BCPS and in 26 (6%) patients at TCPC (Fig. 2).

**Table 3:** Intervention for APCs

Variables		*N* (%)
Number of patients		430
Total		120(27.9)
Timing of intervention		
	Pre-BCPS	12 (2.8)
	At BCPS (surgical)	13 (3.0)
	After BCPS	8 (1.9)
	Pre-TCPC	44 (10.2)
	At TCPC (surgical)	26 (6.0)
	After TCPC	52 (12.1)
Location		
	RIMA	65 (15.1)
	LIMA	33 (7.7)
	Other APCs	74 (17.2)

APCs: aortopulmonary collaterals; BCPS: bidirectional cavopulmonary shunt; LIMA: left internal mammary artery; RIMA: right internal mammary artery; TCPC: total cavopulmonary connection.

### Factors associated with the development of aortopulmonary collaterals

As for the factors associated with the development of APCs before BCPS, HLHS (*P* = 0.001), Norwood procedure (*P* = 0.001), younger age at BCPS (*P* = 0.008) and lower weight at BCPS (*P* = 0.029) were identified using univariable analysis (Table 4). Multivariable analysis demonstrated HLHS (*P* = 0.001) as an independent factor.

**Table 4:** Preoperative variables influencing the development of APCs before BCPS

Variables	Univariable model			Multivariable model		
	*P*-value	OR	95% CI	*P*-value	OR	95% CI
HLHS	**0.001**	2.660	1.483–4.773	**0.001**	2.660	1.483–4.773
TA	0.869	1.070				
DILV	0.791	1.132				
PAIVS	0.264	0.315				
ccTGA	0.339	0.371				
Heterotaxy	0.120	0.203				
TAPVC	0.617	0.731				
Dextrocardia	0.429	0.611				
Dominant RV	0.255	1.418				
Norwood/DKS	**0.001**	2.861	1.526–5.367			
PAB	0.117	0.314				
APS	0.453	0.807				
Pre-BCPS PAP	0.119	0.937				
Pre-BCPS Nakata-Index	0.684	0.992				
Age at BCPS	**0.008**	0.859	0.768–0.960			
Weight at BCPS	**0.029**	0.781	0.626–0.975			
PA reconstruction at BCPS	0.751	1.103				

APCs: aortopulmonary collaterals; APS: aortopulmonary shunt; BCPS: bidirectional cavopulmonary shunt; ccTGA: congenitally corrected transposition of the great arteries; CI: confidence interval; DILV: double inlet left ventricle; DKS: Damus–Kaye–Stansel procedure; HLHS: hypoplastic left heart syndrome; OR: odds ratio; PA: pulmonary artery; PAB: pulmonary artery banding; PAIVS: pulmonary atresia with intact ventricular septum; PAP: pulmonary artery pressure; RV: right ventricle; TA: tricuspid atresia; TAPVC: total anomalous pulmonary venous connection.

The bold values denotes significant in statistical analysis <0.05.

The factors associated with APC development prior to TCPC included Norwood procedure (*P* = 0.045), number of palliative procedures (*P* = 0.047), pre-BCPS lower Nakata-Index (*P* = 0.002), pre-TCPC lower Nakata-Index (*P* = 0.012), pre-TCPC lower systemic ventricular pressure (*P* = 0.009) and pre-TCPC lower aortic mean pressure (*P* = 0.002). These were also identified using univariable analysis (Table 5). Multivariable analysis revealed a lower Nakata-Index pre-BCPS (*P* = 0.026) and younger age at BCPS (*P* = 0.011) as independent factors.

**Table 5:** Preoperative variables influencing development of APCs before TCPC

Variables	Univariable model			Multivariable model		
	*P*-value	OR	95% CI	*P*-value	HR	95% CI
HLHS	0.513	1.146				
TA	0.274	1.358				
DILV	0.745	1.111				
PAIVS	0.726	1.167				
ccTGA	0.983	1.010				
Heterotaxy	0.017	0.352	0.149–0.831			
TAPVC	0.069	0.464				
UAVSD	0.129	0.368				
Dominant RV	0.267	0.802				
Norwood/DKS	**0.045**	**1.483**	**1.008–2.181**			
PAB	0.058	0.510				
APS	0.220	1.243				
Number of palliation	**0.047**	**1.403**	**1.005–1.970**			
Pre-BCPS Nakata-Index	**0.002**	**0.995**	**0.935–0.992**	**0.026**	**0.999**	**0.906–0.994**
Pre-TCPC Nakata-Index	**0.012**	**0.995**	**0.990–0.999**			
Pre-TCPC PAP	0.950	0.998				
Pre-TCPC SVP	**0.009**	**0.978**	**0.962–0.994**			
Pre-TCPC AoP	**0.001**	**0.965**	**0.945–0.987**			
Pre-TCPC SO2	0.656	0.993				
Age at TCPC	**0.049**	**0.899**	**0.808–1.000**			
Interval BCPS and TCPC	0.499	0.958				
Weight at TCPC	0.151	0.973				
Age at BCPS	**0.012**	**0.965**	**0.938–0.992**	**0.011**	**0.874**	**0.787–0.969**
Weight at BCPS	**0.026**	**0.885**	**0.794–0.986 **			

AoP: mean aortic pressure; APCs: aortopulmonary collaterals; APS: aortopulmonary shunt; BCPS: bidirectional cavopulmonary shunt; ccTGA: congenitally corrected transposition of the great arteries; CI: confidence interval; DILV: double inlet left ventricle; DKS: Damus–Kaye–Stansel procedure; HLHS: hypoplastic left heart syndrome; OR: odds ratio; PAB: pulmonary artery banding; PAIVS: pulmonary atresia with intact ventricular septum; PAP: pulmonary artery pressure; RV: right ventricle; SO2: arterial oxygen saturation; SVP: systemic ventricle pressure; TA: tricuspid atresia; TAPVC: total anomalous pulmonary venous connection; TCPC: total cavopulmonary connection.

The bold values denotes significant in statistical analysis <0.05.

### Influence of aortopulmonary collaterals for outcomes after total cavopulmonary connection

As for the factors influencing the survival after TCPC, the presence of APCs before TCPC or intervention for APCs before TCPC did not affect mortality (Supplementary Material, Table S3). Whereas, pre-TCPC pulmonary artery pressure (*P* = 0.023) and pre-TCPC atrioventricular valve regurgitation (*P* = 0.024) were factors associated with mortality after TCPC with univariable analysis. These two variables remained as independent factors with multivariable analysis.

### Sub-group analysis comparing the effects of aortopulmonary collaterals closure in patients with aortopulmonary collaterals

**Figure 3:**
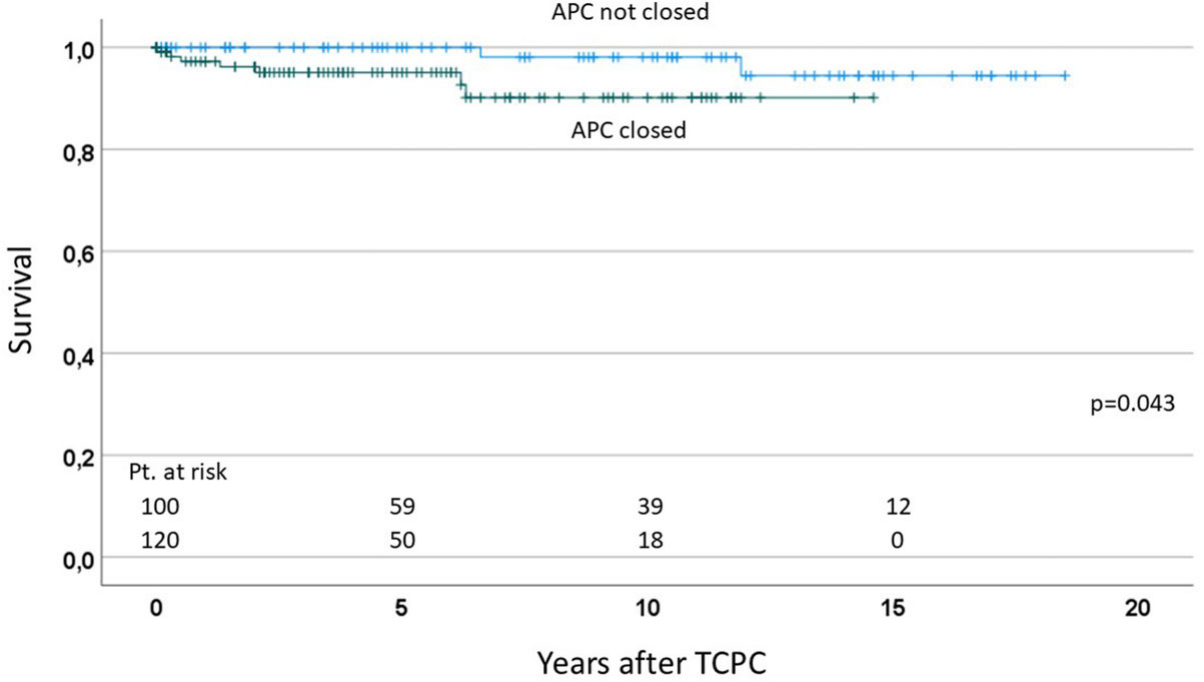
Number of interventions for APCs according to the timing of the procedure. APCs: aortopulmonary collaterals; TCPC: total cavopulmonary connection.

We performed a sub-group analysis comparing the effects of APCs closure in 220 patients who developed APCs. When we compared the 120 patients who underwent APC closure with the remaining 100 patients who could not close their APCs, survival after TCPC was significantly lower in patients who closed their APCs (*P* = 0.043), compared with those who did not (Fig. 3). Freedom from catheter intervention was also lower in patients who closed their APCs, compared with those who did not (*P* < 0.001).

## DISCUSSION

This study revealed that APCs were present at 13% before BCPS and 42% before TCPC. Out of these patients, the presence of APCs was most frequently associated with HLHS. The presence of APCs was associated with lower Nakata-Index at the time of TCPC. Sub-group analysis with 220 patients who developed APCs showed that patients who closed their APCs had lower survival and lower freedom from catheter interventions after TCPC, compared with those who did not.

### Incidence of aortopulmonary collaterals and assessment of aortopulmonary collaterals flow

APCs detected by angiogram have been described in 17–85% of patients after BCPS [1–5]. Previous studies using CMR showed that APC flow was highest in pre-Fontan patients and decreased after the Fontan operation [10]. Whitehead *et al.* [9] demonstrated that APCs do not decrease in the short term after TCPC completion and trend towards increase, but APCs decrease over time after TCPC completion. Our results also demonstrated the incidence of APCs was highest before TCPC and decreased after TCPC.

To assess the APC flow, eye-balling from angiography or the rate of blood flow returning to the heart during aortic cross-clamping at the time of operation were used as the standard method [6, 25]. Recently, CMR imaging techniques emerged as a reliable tool to analyse the physiology and clinical significance of APCs [8, 9]. A previous study compared the CMR results and angiographic findings in the pre-Fontan patients and demonstrated that the APC flow measured by CMR was more sensible than the angiographic APCs grade but showed significant overlap between grades [10].

In this study, APCs were more frequently observed on the right side than the left side. The study by Grosse-Wortmann *et al.* demonstrated in their study using CMR that APC flow was greater to the left lung than to the right lung. Our explanation is that the total volume of pulmonary perfusion area and total lung volume is larger in the right side in most of the patients.

### Factors related to the development of aortopulmonary collaterals

The potential aetiologic factors that promote APC formation are incompletely understood. Previous studies suggested that factors associated with its presence were patient age [1, 25], pulmonary artery size and anatomy [1, 3, 25], arterial oxygen saturation [1, 2, 6, 25] and prior mediastinal or pleural trauma and inflammation [3, 15]. In this study, HLHS, previous Norwood procedure, number of palliations and Nakata-Index were identified as factors associated with the development of APCs. Whether lower PBF promotes the development of APCs or APCs inhibit sufficient development of the pulmonary arteries is not clear. Some studies have shown that turbulent collateral flow impedes effective blood flow, which ends in a loss of energy within the PBF and disrupts blood flow through the arteries and capillaries [11, 26].

### Clinical significance of aortopulmonary collaterals for outcome after total cavopulmonary connection

The clinical significance of APCs after the Fontan procedure remains a topic of discussion and controversy. Several studies in early era showed that APC flow correlated positively with prolonged pleural effusions [4, 5]. Conversely, other studies demonstrated no association between APCs flow and prolonged pleural effusions or haemodynamic parameters after the Fontan procedure [3, 6]. Thus, the amount of APC flow is most likely important. Glatz *et al.* [12, 13] showed that greater amounts of APC flow were more likely to require hospitalization of at least 7 days and chest tube duration of at least 10 days. In theory, APCs provide competitive flow to the lungs and may increase pressure in the systemic venous pathways. Mainwaring *et al.* [27] suggested that accessory PBF represent a ‘steal’ from the systemic circulation and hence may contribute to the incidence of low cardiac output. Therefore, it may be true that considerable APC flow influences the postoperative outcome after the Fontan procedure, although the precise mechanism, through which APCs adversely affect the early postoperative outcomes, is still inadequately understood. In this study, sub-group analysis with 220 patients who developed APCs, patients who closed their APCs had lower survival and lower freedom from catheter interventions after TCPC, compared with those who did not. We assume that relevant APCs, which developed during the staged palliation, had still relevant negative effects on survival after the Fontan procedure in spite of aggressive coil embolization of APCs.

Another concern is whether APCs affect the development of pulmonary arteries or not. Prakash *et al.* [10] demonstrated that patients with unilateral branch pulmonary artery stenosis had higher APC flow. Ichikawa *et al.* [25] showed higher APC flow had low Nakata-Index. Odenwald *et al.* [14] and Wang *et al.* [28] demonstrated a significant negative correlation between APCs flow and Nakata-Index. Our results demonstrated that both lower Nakata-Index pre-BCPS and pre-TCPC were associated with the development of APCs. In patients with unilateral pulmonary stenosis, the increase in PBF by APCs will result in higher arterial oxygenation saturation and thus obviate excessive hypoxaemia. On the other hand, the development of APCs might interfere with the development of pulmonary arteries. Relief of unilateral PA stenosis before Fontan procedure may also contribute to a decrease in APCs. Our experience and previous studies suggest that pulmonary artery growth is satisfactory for Fontan completion regardless of whether APCs are present. Further studies are needed to clarify this issue using precise assessment of serial pulmonary artery size and APCs flow.

### Closure of aortopulmonary collaterals

We routinely perform cardiac catheterization and angiogram before BCPS and before TCPC in all patients. The purpose of cardiac catheterization is to evaluate the haemodynamics (measuring pulmonary artery pressure, atrial pressure, etc.) and to evaluate the development of the pulmonary artery, APCs, PAVMs and Re-coarctation of the aorta. We also check the (ab)normality of systemic venous return (inferior vena cava (IVC) and hepatic veins) before TCPC. Some institutions perform CMR instead of cardiac catheterization before BCPS and/or TCPC. However, we believe that cardiac catheterization is mandatory before BCPS and TCPS, because we needed interventions, such as coil embolizations of APCs, stent implantation for re-coarctation, closure of veno-venous collaterals.

Because APCs increase the volume work of the systemic ventricle and have an adverse effect on pulmonary vasculature, we aggressively perform interventional coil embolization and surgical closure of APCs during each stage of surgical palliation. The closure of APCs may result in an improvement in both myocardial performance and pulmonary vascular resistance, which may confer advantages not only at the time of Fontan but also in the long term. As there is no randomized study in regards to APCs closure, it is difficult to know its effects on outcome after the Fontan procedure. Dori *et al.* [17] demonstrated the acute effects of coil embolization of APCs in patients after BCPS. However, the durability of the manoeuvre and its effect on outcomes after Fontan remained controversial. Some studies demonstrated that APC occlusion improved postoperative outcomes [4, 5]; others showed no association between pre-Fontan coiling of APCs and postoperative outcomes after Fontan procedure [3, 29]. Our results did not confirm the positive effects of APC occlusion. The previous report demonstrated that the coil embolization of APCs after Fontan procedure facilitated Fontan circulation [30], whereas Kanter *et al.* [5] showed that significant presence of APCs after TCPC may be a factor associated with mortality.

### Limitations

This study was limited by its retrospective, non-randomized and single-centre design. The diagnosis of APCs is somewhat arbitrary; the ability to identify them is highly dependent of technical variables and created detection biases. The timing APCs developed might be different from the time at catheterization and also created detection biases. The analysis of the factors associated with APCs may be affected by detection biases and that the inclusion of covariates by purely statistical criteria may lead to the detection of spurious associations of little or no clinical relevance. This study used the angiographic appearance of APCs as a surrogate for the amount of APC flow. Therefore, haemodynamic load could not be quantified and lack of direct measurement of APCs flow by CMR is a substantial limitation.

## CONCLUSIONS

In 430 patients, who underwent staged Fontan completion, APCs were most frequently observed before TCPC. HLHS was a significant factor associated with the development of APCs before BCPS. Patients who demonstrated with APCs before TCPC had significantly lower Nakata-Index, but presence of APCs before TCPC did not affect the postoperative outcomes after TCPC. Sub-group analysis with patients who demonstrated APCs during the staged palliation, patients who could close their APCs before Fontan procedure demonstrated worse survival and higher incidence of catheter interventions after TCPC. These results augmented that relevant APCs which needed interventions during the staged palliation affect the post-Fontan outcomes.

## SUPPLEMENTARY MATERIAL

Supplementary material is available at *ICVTS* online.

### Funding

Dr. Takashi Kido was supported by the Förderverein des Deutschen Herzzentrums München.

**Conflict of interest:** The authors declare no potential conflicts of interest with respect to the research, authorship, or publication of this article.

## Supplementary Material

ivac190_Supplementary_Data

## Data Availability

The data underlying this article will be shared on reasonable request to the corresponding author.

## Notes

Presented at the 51st Annual Meeting of the German Society of Thoracic and Cardiovascular Surgery, 18–20 February 2022 held at Hamburg, Germany.

## ABBREVIATIONS

APCs: Aortopulmonary collaterals
BCPS: Bidirectional cavopulmonary shunt
CMR: Cardiac magnetic resonance
HLHS: Hypoplastic left heart syndrome
LIMA: Left internal mammary artery
PBF: Pulmonary blood flow
RIMA: Right internal mammary artery
TCPC: Total cavopulmonary connection
