# Supplementary figures and images for: Aortopulmonary collaterals in single ventricle: incidence, associated factors and clinical significance
Source: Interact Cardiovasc Thorac Surg. 2022 Jul 25;35(2):ivac190. doi: 10.1093/icvts/ivac190 (PMC9318886; doi:10.1093/icvts/ivac190)

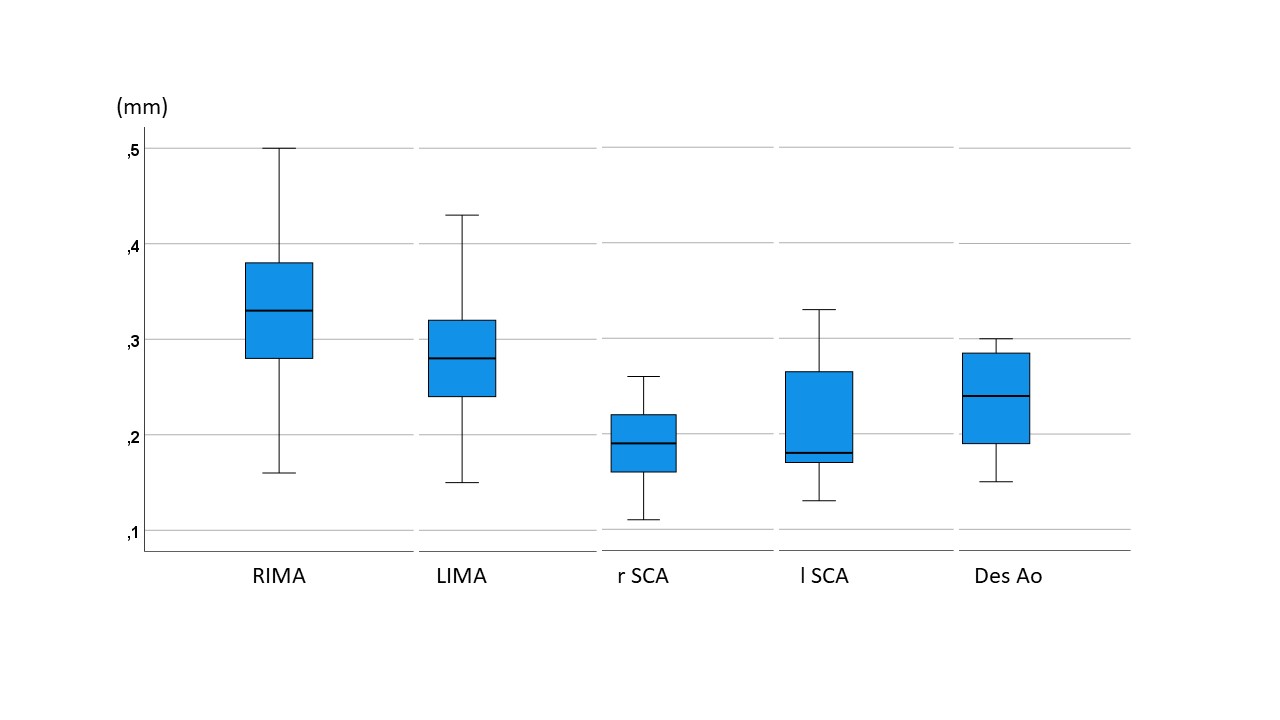

Supplement: ivac190_Supplementary_Data [file ivac190_supplementary_data.zip › SupFigure2_SchmielAPCsSV_12022022.jpg]

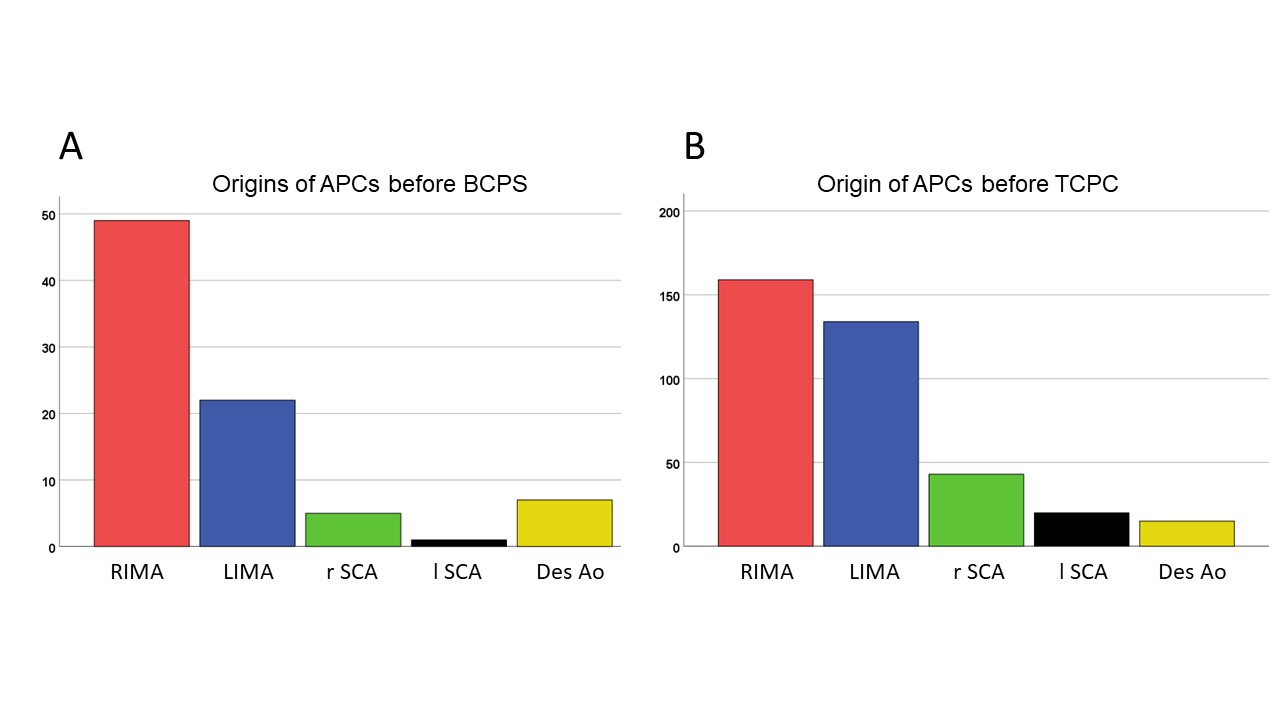

Supplement: ivac190_Supplementary_Data [file ivac190_supplementary_data.zip › SupFigure1_SchmielAPCsSV_12022022.jpg]
